# Weight Change and Its Association with Cardiometabolic Risk Markers in Overweight and Obese Women

**DOI:** 10.1155/2020/3198326

**Published:** 2020-04-13

**Authors:** Liyana Ahmad Zamri, Geeta Appannah, Siti Yazmin Zahari Sham, Fazliana Mansor, Rashidah Ambak, Noor Safiza Mohd Nor, Tahir Aris

**Affiliations:** ^1^Endocrine and Metabolic Unit, Institute for Medical Research, National Institutes of Health, Ministry of Health Malaysia, Setia Alam, 40170 Shah Alam, Selangor, Malaysia; ^2^Department of Nutrition and Dietetics, Faculty of Medicine and Health Sciences, Universiti Putra Malaysia, 43400 Serdang, Selangor, Malaysia; ^3^Department of Pathology, Faculty of Medicine and Health Sciences, Universiti Putra Malaysia, 43400 Serdang, Selangor, Malaysia; ^4^Institute for Public Health, National Institutes of Health, Ministry of Health Malaysia, Setia Alam, 40170 Shah Alam, Selangor, Malaysia; ^5^Allied Health Sciences Division, Ministry of Health Malaysia, 62050 Putrajaya, Malaysia

## Abstract

**Objectives:**

To examine the association of weight loss magnitude with changes in cardiometabolic risk markers in overweight and obese women from low socioeconomic areas engaged in a lifestyle intervention.

**Methods:**

Analyses were performed on 243 women (mean body mass index 31.27 ± 4.14 kg/m^2^) who completed a 12-month lifestyle intervention in low socioeconomic communities in Klang Valley, Malaysia. Analysis of covariance (ANCOVA) was used to compare changes of cardiometabolic risk factors across weight change categories (2% gain, ±2% maintain, >2 to <5% loss, and 5 to 20% loss) within intervention and control group.

**Results:**

A graded association for changes in waist circumference, fasting insulin, and total cholesterol (*p*=0.002, for all variables) across the weight change categories were observed within the intervention group at six months postintervention. Participants who lost 5 to 20% of weight had the greatest improvements in those risk markers (−5.67 cm CI: −7.98 to −3.36, −4.27 *μ*U/mL CI: −7.35, −1.19, and −0.59 mmol/L CI: −.99, −0.19, respectively) compared to those who did not. Those who lost >2% to <5% weight reduced more waist circumference (−4.24 cm CI: −5.44 to −3.04) and fasting insulin (−0.36 *μ*U/mL CI: −1.95 to 1.24) than those who maintained or gained weight. No significant association was detected in changes of risk markers across the weight change categories within the control group except for waist circumference and adiponectin.

**Conclusion:**

Weight loss of >2 to <5% obtained through lifestyle intervention may represent a reasonable initial weight loss target for women in the low socioeconomic community as it led to improvements in selected risk markers, particularly of diabetes risk.

## 1. Introduction

Obesity has become a significant public health threat around the globe. Despite reducing the quality of life [1], obesity is associated with noncommunicable diseases, including diabetes mellitus, coronary heart disease, stroke, and several types of cancers [2, 3] as well as mortality [4]. Malaysia has been battling with the problem for more than a decade, whereby the prevalence of obesity in the adult population ranked as the highest in South East Asia [5]. The problem also disproportionately affected women, particularly those from a lower socioeconomic status.

Body weight loss of at least 3% from the baseline level is considered as clinically relevant as such amount associated with improvements in multiple cardiometabolic risk markers [6, 7]. As reported recently, overweight/obese adults who underwent 8 months of exercise training with at least 3% of weight loss significantly improved their insulin sensitivity, acute insulin response, triglycerides, non-HDL cholesterol concentration, low-density lipoprotein (LDL) particle size, and high-density lipoprotein (HDL) particle size [8]. The findings were in line with previous studies [9, 10] which demonstrated improvement in several cardiometabolic risk markers with modest weight loss of ≥3%. Additionally, PODOSA (Prevention of Diabetes and Obesity in South Asians) trial showed that each 1 kg weight reduction, an achievable amount of weight loss, was significantly associated with reductions in triglycerides, ALT, GGT, leptin, insulin, fasting glucose, 2-hour glucose, and HOMA-IR [11].

However, the data regarding weight loss intervention and its effect on cardiometabolic risk markers among overweight and obese women in the low socioeconomic community are scant. Tackling the obesity problem among low-income women presents unique challenges that warrant attention if the weight loss interventions are to be effective. The most common perceived barriers to healthy eating and physical activity encountered by these women were related to cost [12], motivation, and support from spouse and family [13], time and cultural issues [14] as well as safety concerns [15]. Examining the data by weight change interval will determine a proportion of individuals in a lifestyle intervention who achieved a clinically relevant weight loss even though the average weight loss is small or modest [16]. Additionally, promoting weight loss among obese women of low-income was proven difficult with some reported small or nonsignificant weight loss [17, 18].

Hence, the purpose of the present study was to (1) determine the overall prevalence of participants achieving weight loss of 5 to 10%, weight loss of >2 to <5%, weight maintained (±2%) and weight gained (>2%) among overweight and obese women following a 6-month lifestyle intervention and a 6-month maintenance period; (2) examine the correlations between per cent weight loss and change in cardiometabolic risk markers after 12 months; and (3) compare the mean changes of those markers in women who achieved 5 to 20% weight loss to those who did not in both study groups.

## 2. Materials and Methods

### 2.1. Study Design and Population

This study utilised data from a lifestyle intervention among overweight and obese women in low socioeconomic areas in Klang Valley, namely, the My Body is Fit and Fabulous at home (MyBFF@home) conducted from the year 2013 to 2015. Information on study design and recruitment details were published elsewhere [19]. Briefly, MyBFF@home was a quasiexperimental study comparing women who received an intervention package to the control group (*N* = 243). The study was divided into two phases: weight loss intervention phase (first 6 months) and maintenance phase (6–12 months). During the weight loss intervention phase, participants attended monthly 1-hour individual diet counselling and moderate physical activities (in a group format), separately. The participants were advised on a reduced-calorie diet (1200–1500 kcal/day) through education on food labelling, portion control, and food substitution (low fat and low sugar choices). They were also encouraged to do moderate physical activities, 7 days/week. Self-monitoring tools such as pedometer, 3-day food diary, and 3-day physical activity diary with MET calendar were given to the participants to monitor their diet and physical activities. During the maintenance phase, these activities were not supervised by the study researchers, and the frequency of contact was reduced to 2 sessions (at months 9 and 12). The control group received general women's health seminars during the follow-up sessions. The participants were recruited based on the following criteria: (i) body mass index (BMI): 25 to 39.99 kg/m^2^, (ii) age: 18 to 59 years, (iii) not working/housewives, and (iv) able to speak Malay/English. Those who were morbidly obese (≥40 kg/m^2^), pregnant, currently on weight management regime, or had physical disability and comorbidities (i.e., hypertension, diabetes, renal dysfunction) were excluded from the study. Anthropometric measurements and cardiometabolic risk markers were assessed at baseline, 6, and 12 months and subsequently evaluated by study phases (i.e., weight loss intervention phase (baseline to 6 months) and weight maintenance phase (6 to 12 months)). All study participants provided written informed consent before the study conduct. The research protocol was approved by the Malaysian Research Ethics Committee (MREC), trial no. NMRR-13-726-16391.

### 2.2. Anthropometric Assessment

Weight was measured in light clothing and without shoes using a digital scale in kilograms (kg) (Tanita HD319, Japan). Waist circumference (WC) was measured in centimetres (cm) at the midpoint between the lower rib and the iliac crest in triplicate. Measurement details were previously reported [19].

### 2.3. Cardiometabolic Risk Markers Assessment

Cardiometabolic risk markers assessed in this study including fasting plasma glucose (FPG), fasting insulin, HOMA-IR as an indicator for insulin resistance, lipid profiles, and adipokines such as adiponectin, high sensitivity C-Reactive Protein (hs-CRP), and tumour necrosis factor-alpha (TNF-*α*). Venous blood samples were collected after 10 hours of fasting. FPG, TC, HDL-C, LDL-C, and triglycerides were analysed on clinical chemistry analyser (CS-400 Dirui, China) using appropriate reagents, calibrators, and controls (Randox Laboratories, UK). Plasma insulin was measured using TOSOH AIA-360 system analyser (Tosoh Corporation, Japan). Insulin resistance was assessed using the homeostasis model assessment of insulin resistance (HOMA-IR) which is calculated as fasting glucose (mmol/L) × insulin (*μ*U/mL)/22.5 [20]. The serum levels of adiponectin and TNF-*α* were measured by enzyme-linked immunosorbent assay (ELISA) (Quantikine ELISA, R&D system, Minneapolis, USA) according to the manufacturer's instruction. hs-CRP levels were determined by a high sensitivity ELISA (IBL International GMBH, Hamburg, Germany).

### 2.4. Statistical Analysis

Descriptive data were tabulated as means ± standard deviation (SD) or 95% confidence intervals (95% CI) as appropriate. The per cent weight change was calculated as weight at time 2–weight at time (1)/weight at time 1 × 100%. The participants in both study groups were stratified into four weight change categories: (1) >2% gain, (2) ±2% maintain, (3) >2 to <5% loss, and (4) 5 to 20% loss of initial body weight [21]. Due to the small number of participants who lost >10 to 20% of initial weight (i.e., less than 5 participants in each study phase), the category was combined with those who had 5 to 10% weight loss in a single category (5 to 20%).

The baseline characteristics across the weight change category were compared using analysis of variance (ANOVA), and statistically different variables were used as covariates in the later analysis. The distribution of participants in the intervention and control group by weight change category were compared using the chi-square test. The correlations between the magnitude of weight loss and cardiometabolic risk markers were assessed using Spearman's (nonadjusted) and partial correlation (controlling for age, baseline weight, and baseline measurement of variable outcomes) by study groups.

The mean changes in the outcome variables were stratified by weight change category, and the differences were evaluated using analysis of covariance (ANCOVA) within intervention and control groups. The covariates within the statistical model included age, baseline weight, and baseline measurements of variable outcomes. The ANCOVA analyses were verified for the equal group variance, independence of covariate and treatment effects, and homogeneity of regression slopes. The results were presented in estimated marginal means with 95% CI. All statistical analyses were conducted using Statistical Package for Social Science (SPSS) for Windows© version 22.0 (Chicago, IL, US). The analyses were tested at two-sided, and the significance level was set at *p* < 0.05.

## 3. Results

### 3.1. Baseline Characteristics of Participants by Weight Change Category

Table 1 presents the baseline characteristics of participants in both study groups according to weight change after 12 months. One-way ANOVA revealed that there were no statistically significant differences in baseline mean of outcome variables across the weight change category in either the intervention or control group except for fasting insulin, HOMA-IR, adiponectin, and hs-CRP in the intervention group. Those who gained >2% of initial weight showed higher baseline levels of adiponectin and hs-CRP. In contrast, individuals who lost 5 to 20% of weight had higher baseline levels for fasting insulin and HOMA-IR. The baseline levels of these variables were considered as covariates in the later analyses.

### 3.2. Distribution of Participants by Weight Change Category

**Figure 1:**
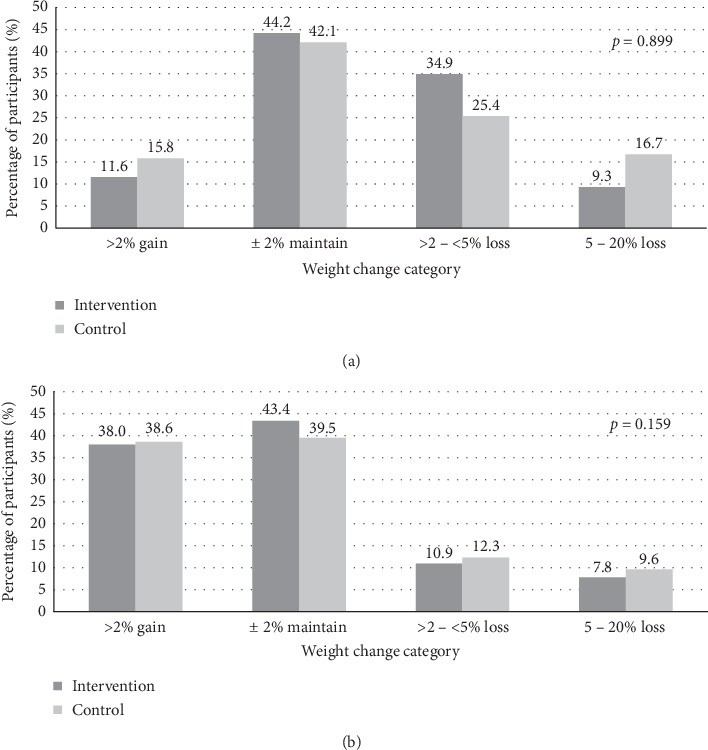
The proportion of participants in the intervention (*n* = 129) and control (*n* = 114) groups according to the weight change category during (a) weight loss intervention phase and (b) maintenance phase. *Note.p* value indicates the statistical significance of the chi-square test used to examine the between-group difference in the proportions of participants with different weight change categories, significant at *p* < 0.05.

The distribution of participants in each weight loss category was summarised in Figure 1. More participants in the control group lost between 5 to 20% weight at 6 months postintervention and 12 months after the maintenance period end compared to the participants in the intervention group (i.e., 16.7% vs. 9.3% and 9.6% vs. 7.8%, respectively). Nevertheless, the intervention group showed a higher proportion of those who lost between >2% to <5% and maintained ±2% of weight (i.e., 34.9% vs. 25.4% and 44.2% vs. 42.1%, respectively) and less weight gained (11.6% vs. 15.8%) than the control group at 6 months.

However, the proportion of those losing >2 to <5% and 5 to 20% weight dropped by 1.5% and 7.1% in the intervention and control groups, respectively, during the weight maintenance phase. While the proportion of weight gained >2% increased approximately threefold in both study groups, only a slight decrease was observed in the proportion of participants who maintained their weight during this phase. However, the chi-square test revealed no statistically significant differences across weight loss categories between study groups in both study phases.

### 3.3. Correlation of Change in Cardiometabolic Risk Markers with Percent Weight Loss after 12 Months

Correlations of the magnitude of changes between weight in percentage and cardiometabolic risk markers after 12 months were presented in Table 2. In the intervention group, change in weight positively correlated to changes in WC (*r* = 0.366), fasting insulin (*r* = 0.228), HOMA-IR (*r* = 0.270), and FPG (*r* = 0.228) after adjusting for covariates, whereas weak correlations were found for changes in other lipid measures and inflammatory markers. Significant correlations were also observed in the control group between change in weight and changes in WC, fasting insulin, and HOMA-IR (0.218 ≤ |*r*| ≤ 0.278). However, the magnitude of these correlations was smaller compared to the intervention group. The changes in other risk markers showed a weak correlation to change in weight.

### 3.4. Association of Weight Change with Improvement in Cardiometabolic Risk Markers during the Weight Loss Intervention Phase

Table 3 demonstrates the mean changes in cardiometabolic risk markers according to categories of weight change during the weight loss intervention phase. A significant association for changes in WC, fasting insulin, and TC (*p*=0.002, for all variables) across the weight change categories were observed within the intervention group where participants who lost 5 to 20% weight showed the most favourable changes compared to other weight change categories. In direct comparison, improvements in WC and fasting insulin of participants who lost >2% to <5% were greater than those who maintained ±2% weight and gained >2% weight, albeit insignificant difference between the categories. In contrast, the magnitude of improvements in other markers did not significantly differ across the weight change categories.

Similarly, the control group exhibited a graded association for changes in WC where more reduction in WC occurred with higher weight loss. Those who lost 5 to 20% of initial weight increased adiponectin levels substantially than those who gained or maintained weight (*p* < 0.001). No significant association was detected in changes of other markers across the weight change categories.

### 3.5. Association of Weight Change with Improvement in Cardiometabolic Risk Markers during the Maintenance Phase

The mean changes in cardiometabolic risk markers across categories of weight change during the weight loss maintenance phase were presented in Table 4. Both study groups showed a significant graded association between weight change and improvement in WC (*p* < 0.01 for both groups). Thereby participants who lost between >2 and <5% and 5 to 20% weight had more WC reduction than those who maintained and gained weight, whereas no significant differences were found in mean changes of other risk markers when compared across the weight change categories during this phase. While in the control group, there was a significant difference between mean changes in HDL-C (*p*=0.018), the improvement was observed in those who gained weight compared to the other weight change categories.

## 4. Discussion

The present study showed that the majority of participants either lost >2 to <5% of weight or maintained their weight (±2% of initial weight) following a 6-month lifestyle intervention, and the proportion was higher than that in the control group. Although such an amount of weight loss was trivial, it was observed that the lifestyle intervention assessed in this study may have prevented further weight gain. Prevention of weight gain is equally essential in a weight loss programme which not only may attenuate the obesity rate but also reduce the risk of chronic diseases including T2DM, CVD, cancer, and nontraumatic death [22]. However, the short-term effects of weight loss were not sustained during the weight maintenance phase.

Of interest, participants in the control group showed improvement in weight whereby the proportions of those losing 5 to 20% weight were relatively higher in both study phases compared to the participants in the intervention group. Unanticipated improvement in the control group was common in a controlled intervention study as individuals who are willing to participate often have a strong desire and motivation to change their habits [23]. As a matter of fact, findings from the main paper of this study [19] found that over 70% of participants in both the groups attempted weight reduction prior to the intervention through multiple ways including fasting, exercising, taking slimming pills and herbs, and controlling diet. Hence, the control group could be contaminated with individuals who are health-conscious and become motivated with subsequent follow-up by the study researchers during data collection (i.e., weight, food, and physical activity diaries). These findings also indicate that health awareness and subsequent weight-monitoring may be adequate to trigger behavioural changes among individuals in a socioeconomically disadvantaged community.

Nonetheless, the proportion of individuals achieving at least 5% weight loss in the present study was lower compared to other intervention studies among overweight and obese women in the low-income community ranging from 19% to almost 50% [24–26]. Those studies emphasised on overall excellence follow-up, adherence to the interventions, acceptability, and feasibility of the intervention that contributed to successful weight loss. Unlike previous studies, the intervention package in this study was less intense as the participants were mostly homemakers and occupied with house chores and other family related commitments. Additionally, the intervention was lack of behavioural component to promote substantial weight loss and this could be considered in designing future studies [27].

The magnitude of weight change after one-year was found significantly correlated to WC, fasting insulin, HOMA-IR, FPG, and triglycerides in the intervention group. The findings suggest that changes in those markers can be attributed to weight loss induced through lifestyle intervention. Significant correlations were also observed in the control group between change in weight and improvements in WC, fasting insulin, and HOMA-IR. The correlations within the control group were expected due to similar average weight loss to the intervention group (−0.10 kg vs. −0.11 kg, respectively) after one year as reported in the main study [28], whereas no significant correlation was detected for changes in other lipids and inflammatory markers with weight loss.

Furthermore, a significant graded association was found between weight loss and changes in WC, fasting insulin, and TC following six months of intervention but the association disappeared during the weight maintenance phase. More reduction was seen in WC as more weight loss occurred and this clear association was found in both study groups. Interestingly, WC reduced in each weight loss category during both study phases. Also, those who lost >2 to <5% of weight reduced WC by more than 3 cm which is likely to alleviate the aggregation of risk factors that contribute to metabolic syndrome [29]. The findings suggest significant health benefits over the one-year course to women in this cohort as they had an abdominal obesity problem and by reducing WC may lower the risk of CVD and T2DM [30],whereas more reduction in fasting insulin level occurred in those who lost at least >2% weight compared to those who maintained or gained weight in the intervention group. Previous studies among overweight and obese postmenopausal women have shown a significant dose-response relationship in changes of FPG, fasting insulin, and HOMA-IR with percentage weight loss following one-year of reduced-calorie programme and/or moderate-to-vigorous intensity aerobic activity [31]. The study also showed improvements in the risk markers even within <5% weight loss group. Significant improvements in HOMA-IR were also reported in overweight and obese women who lost 3 to 4.9% weight (−0.48) and obtained a clinically significant weight loss ≥ 5% (−0.60) after six months of aerobic training [32]. The findings were comparable to the present study, although no significant association was found in the changes of HOMA-IR and weight loss in the intervention group.

For lipid profiles, only TC showed a significant association with weight loss during the weight loss intervention phase within the intervention group. TC was greatly reduced (−0.59 mmol/L 95% CI: −0.99 to −0.19) in individuals who lost between 5 to 20% compared to other weight change categories. Conversely, Swift et al. [32] reported no differences in TC level across the weight change categories among postmenopausal overweight and obese women participating in a 6-month exercise training. In a weight loss trial among overweight and obese women [33], TC level was reduced regardless of the amount of weight loss achieved where the participants had elevated baseline levels whereas LDL-C and triglycerides were improved only in those who lost >10% weight. A systematic review [34] included lifestyle interventions among adults with baseline BMI < 35 kg/m^2^ showed significant beneficial changes in lipid profiles following modest weight loss at 2 to 3 years follow-up. However, the review concluded that the association diminished over the long term suggesting other lifestyle factors such as dietary fat intake, per se caloric restriction, and physical activity that also need to be sustained.

There are several limitations to the present study worth mentioning. The present study utilised data from a quasiexperimental study which does not involve randomisation. Although the study design offers the feasibility to conduct in the community, it may have introduced a bias in selection and limits external validity. Nevertheless, no compelling differences were observed in the baseline characteristics and most of the variables outcomes between the study groups [35]. The baseline differences in some of the markers were taken into account in the analysis.

Also, although the study locations of the intervention and control groups were separated geographically (intervention group at north and control group at the south of Klang Valley), there may be a chance of control contamination as the radius of the study locations were just below 30 kilometres. To limit the contamination between the groups, participants were followed up at their respective residences on different days. The present study included women living in *Projek Perumahan Rakyat/Awam* (PPR/PPA), a low socioeconomic community in Klang Valley which were predominantly Malay and were healthy upon participation in the intervention. Therefore, the findings were unable to be generalised to other populations of low-income women residing in different geographic locations. This study was also not powered to detect significant differences between groups in cardiometabolic risk markers as it was in accordance with the main study (i.e., to detect significant differences in body weight). Hence, the insignificant relationships between weight changes and improvements in some of the cardiometabolic risk markers should be interpreted with caution.

Though it was clearly shown that the degree of improvement in selected risk markers was related to the magnitude of weight loss, other factors such as changes in diet and physical activity may also result in positive impacts on those risk markers. However, it is impossible to discriminate the effects of weight loss per se with other impacts from the intervention components in the programme promoted. The present study focused on weight change per se to address the benefits of clinically relevant weight loss on cardiometabolic risk markers. However, other indicators of adiposity including body fat mass and visceral fat mass may have relationships with the magnitude of weight loss and more relevant to changes in cardiometabolic risk markers.

The strengths of the present study include the first weight loss intervention study focusing on low socioeconomic women, a group with increasing rates of obesity as well as having a challenging environment for a healthy lifestyle. Furthermore, the findings owing to limited obesity research among adults in Malaysia whereby most studies were conducted as cross-sectional, qualitative, and systematic reviews and only a small number of intervention studies were currently published [36]. Perhaps, evaluating the association of the magnitude of weight loss to changes in cardiometabolic risk markers occurring within a tailored weight loss intervention may suggest the benefits of modest weight loss to cardiometabolic health among these women.

## 5. Conclusion

The findings of the present study suggest that weight loss of >2% to <5% obtained through a short-term lifestyle intervention could improve risk markers including WC and glucose homeostasis in overweight and obese women of low socioeconomic level. The findings also reaffirm the benefits of a more achievable weight loss target to encourage women in this group to change their lifestyle to a healthier one.

## Figures and Tables

**Table 1:** Baseline characteristics of participants in the control and intervention group stratified by weight change category after 12 months.

Cardiometabolic risk markers	Control					Intervention				
	>2% gain	±2% maintain	>2–<5% loss	5–20% loss	*p* value	>2% gain	±2% maintain	>2–<5% loss	5–20% loss	*p* value
*N*	35	43	19	17		27	58	27	17	
Age, year	41.17	42.26	43.74	41.00	0.667	40.74	42.95	42.22	43.29	0.640
	(8.09)	(7.80)	(7.12)	(9.31)		(8.83)	(8.12)	(5.85)	(8.59)	
Weight, kg	71.97	71.54	76.30	70.31	0.337	76.35	77.08	72.94	79.83	0.235
	(12.99)	(9.25)	(12.44)	(10.99)		(11.54)	(9.88)	(10.96)	(15.31)	
BMI, kg/m^2^	30.83	30.78	32.23	29.20	0.179	31.78	32.04	30.12	32.83	0.133
	(4.44)	(3.52)	(4.59)	(3.91)		(3.99)	(4.12)	(3.86)	(4.56)	
WC, cm	90.53	93.07	95.97	90.66	0.160	96.08	94.64	93.04	98.86	0.289
	(10.34)	(7.32)	(11.64)	(6.91)		(10.78)	(9.47)	(9.23)	(12.73)	
FPG, mmol/L	5.50	5.48	6.16	5.72	0.328	5.55	5.42	5.77	6.21	0.474
	(0.64)	(0.85)	(1.66)	(1.11)		(0.75)	(0.60)	(2.20)	(2.48)	
Insulin, *μ*U/mL	7.92	9.87	10.37	9.77	0.351	10.61	9.17	9.15	15.03	**0.043**
	(1.61)	(1.66)	(1.78)	(1.94)		(1.83)	(1.90)	(1.89)	(1.64)	
HOMA-IR	1.94	2.37	2.93	2.46	0.229	2.83	2.19	2.26	4.02	**0.019**
	(1.70)	(1.75)	(2.01)	(2.12)		(2.03)	(2.00)	(1.95)	(1.92)	
TC, mmol/L	5.56	5.72	6.05	5.50	0.338	5.74	5.62	5.33	5.66	0.398^§^
	(1.10)	(1.16)	(0.82)	(1.15)		(1.06)	(0.86)	(0.95)	(1.46)	
LDL-C, mmol/L	4.47	4.64	4.94	4.35	0.527	4.63	4.50	4.13	4.42	0.394
	(1.29)	(1.42)	(1.28)	(1.20)		(1.43)	(0.88)	(1.00)	(1.41)	
HDL-C, mmol/L	1.36	1.37	1.42	1.43	0.699	1.32	1.33	1.30	1.26	0.674
	(0.26)	(0.22)	(0.21)	(0.23)		(0.25)	(0.21)	(0.24)	(0.26)	
Triglycerides, mmol/L	1.21	1.36	1.70	1.21	0.052	1.47	1.23	1.38	1.52	0.283
	(0.71)	(0.63)	(0.56)	(0.62)		(0.66)	(0.45)	(0.86)	(0.95)	
TNF-*α*, *μ*g/mL	10.81	11.36	10.65	8.88	0.294	11.59	10.53	10.40	11.41	0.459
	(2.52)	(3.57)	(3.78)	(3.68)		(3.12)	(2.56)	(1.84)	(3.83)	
Adiponectin, *μ*g/mL^†^	6.27	6.41	7.20	13.74	0.092	10.27	5.81	4.29	4.02	**<0.001**
	(1.54)	(2.36)	(3.15)	(2.48)		(1.99)	(2.03)	(1.95)	(1.41)	
Hs-CRP, mg/L^†^	2.37	3.16	4.17	3.99	0.341	5.88	3.64	1.77	3.68	**0.002**
	(2.12)	(3.13)	(1.81)	(2.33)		(3.64)	(2.29)	(3.17)	(2.03)	

*Note.* Data were presented as mean ± SD. *p* value indicates the statistical significance of the ANOVA test for comparison between weight change categories. Values in bold indicate statistical significance at *p* < 0.05.

**Table 2:** Correlations between changes in cardiometabolic risk markers and per cent weight change over 12 months.

	*n*	Δweight (in percentage)			
		Control		Intervention	
		Unadjusted	Adjusted^b^	Unadjusted	Adjusted^b^
ΔWC, cm	243	**0.341** ^*∗∗∗*^	**0.278** ^*∗∗*^	**0.370** ^*∗∗∗*^	**0.366** ^*∗∗∗*^
ΔFPG, mmol/L	243	−0.033	−0.076	0.144	**0.228** ^*∗*^
ΔFasting insulin, *μ*U/L^a^	221	**0.254** ^*∗*^	**0.218** ^*∗*^	**0.301** ^*∗∗*^	**0.280** ^*∗∗*^
ΔHOMA-IR^a^	212	0.174	**0.225** ^*∗*^	**0.233** ^*∗*^	**0.270** ^*∗∗*^
ΔTriglycerides, mmol/L	243	0.112	0.083	0.092	0.144
ΔTC, mmol/L	243	0.033	0.021	−0.004	0.040
ΔHDL-C, mmol/L	243	0.173	0.131	−0.011	−0.038
ΔLDL-C, mmol/L	243	0.093	0.066	−0.060	−0.021
ΔAdiponectin^,^*μ*g/mL^a^	149	0.103	0.056	0.107	0.145
ΔHs-CRP^,^ mg/L^a^	149	**0.313** ^*∗∗*^	0.134	−0.087	−0.101
ΔTNF-*α*, pg/mL	149	−0.010	−0.029	−0.096	0.014

*Note.* Data were presented as correlation coefficient (*r*). Δ indicates a change in the variables between baseline to 12 months.^a^data were log-transformed prior analysis. ^b^Spearman's partial correlation was adjusted for age and baseline weight. Values in bold indicate statistical significance at ^*∗*^*p* < 0.05, ^*∗∗*^*p* < 0.01, ^*∗∗∗*^*p* < 0.001.

**Table 3:** Mean changes in cardiometabolic risk factors by weight change category in the weight loss intervention phase.

Cardiometabolic risk markers	Weight loss intervention phase (baseline to 6 months)									
	Control					Intervention				
	>2% gain	±2% maintain	>2–<5% loss	5–20% loss	*p* value	>2% gain	±2% maintain	>2–<5% loss	5–20% loss	*p*-value
	*n* = 18	*n* = 48	*n* = 29	*n* = 19		*n* = 15	*n* = 57	*n* = 45	*n* = 12	
WC, cm	−1.27	−1.88	−3.51	−7.63^abc^	**<0.001**	−0.77	−2.14	−4.24	−5.67^a^	**0.002**
	(−3.70, 1.16)	(−3.35, −0.41)	(−5.39, −1.62)	(−9.96, −5.29)		(−2.86, 1.31)	(−3.20, −1.08)	(−5.44, −3.04)	(−7.98, −3.36)	
FPG, mmol/L	−0.37	0.09	−0.05	−0.12	0.434	−0.03	0.07	−0.35	−0.19	0.065
	(−0.85, 0.10)	(−0.20, 0.38)	(−0.42, 0.33)	(−0.59, 0.34)		(−0.08, 0.38)	(−0.48, 0.27)	(−0.33, 0.16)	(−0.67, 0.03)	
Fasting insulin, *μ*U/mL^*∗*^	0.79	0.37	−0.38	−1.32	0.346	−1.08	0.99	−0.36	−4.27^a^	**0.022**
	(−0.34, 0.77)	(−0.79, 1.52)	(−1.86, 1.10)	(−3.19, 0.54)		(−3.91, 1.74)	(−0.43, 2.41)	(−1.95, 1.24)	(−7.35, −1.19)	
HOMA-IR^*∗*^	0.22	0.17	−0.17	−0.45	0.218	−0.55	0.07	−0.48	−0.63	0.190
	(−0.66, 1.84)	(−0.19, 0.53)	(−0.63, 0.29)	(−1.02, 0.12)		(−1.34, 0.24)	(−0.33, 0.46)	(−0.92, −0.03)	(−1.48, 0.23)	
Triglycerides^,^ mmol/L	0.02	−0.01	0.28	0.11	0.253	0.14	0.18	0.07	−0.25	0.166
	(−0.28, 0.31)	(−0.19, 0.17)	(0.05, 0.51)	(−0.18, 0.40)		(−0.17, 0.45)	(0.02, 0.34)	(−0.11, 0.25)	(−0.60, 0.10)	
TC, mmol/L	−0.10	−0.32	−0.26	−0.32	0.760	−0.31	−0.30	0.03	−0.59	**0.022**
	(−0.45, 0.24)	(−0.53, −0.11)	(−0.53, 0.01)	(−0.65, 0.02)		(−0.67, 0.06)	(−0.48, −0.11)	(−0.18, 0.24)	(−0.99, −0.19)	
HDL-C, mmol/L	−0.04	−0.04	−0.09	−0.05	0.674	−0.04	0.01	−0.01	−0.01	0.880
	(−0.13, 0.06)	−0.10, 0.02)	(−0.17, −0.02)	(−0.14, 0.04)		(−0.15, 0.07)	(−0.05, 0.07)	(−0.08, 0.05)	(−0.13, 0.12)	
LDL-C, mmol/L	−0.35	−0.02	−0.07	−0.37	0.412	−0.23	−0.11	0.06	0.08	0.563
	(−0.79, 0.10)	(−0.29, 0.26)	(−0.42, 0.28)	(−0.81, 0.06)		(−0.65, 0.20)	(−0.33, 0.10)	(−0.19, 0.30)	(−0.39, 0.55)	
TNF-*α*, pg/mL^*∗*^	2.76	4.52	4.50	3.66	0.613	0.23	2.43	4.15	5.65	0.182
	(0.44, 5.09)	(3.04, 6.00)	(2.62, 6.37)	(1.35, 5.98)		(−3.78, 4.25)	(0.45, 4.42)	(2.04, 6.26)	(1.80, 9.49)	
Adiponectin, *μ*g/mL^*∗*^	0.89	−2.11	−1.81	5.46^ab^	**<0.001**	1.94	1.86	−0.30	−1.75	0.403
	(−1.87, 3.64)	(−3.93, −0.28)	(−3.91, 0.28)	(2.48, 8.43)		(−2.58, 6.47)	(−0.22, 3.94)	(−2.61, 2.01)	(−5.81, 2.32)	
Hs-CRP, mg/L^*∗*^	1.92	0.62	0.74	0.58	0.779	0.83	−1.15	−1.57	0.60	0.110
	(−0.33, 4.16)	(−0.79, 2.03)	(−0.99, 2.47)	(−1.49, 2.65)		(−1.37, 3.03)	(−2.21, −0.09)	(−2.72, −0.43)	(−1.48, 2.68)	

*Note*. Data were presented as estimated marginal mean (95% CI). ^a^Significant difference as relative to >2% weight gain. ^b^significant difference as relative to ±2% weight maintain. ^c^significant difference as relative to >2–<5% weight loss. ^*∗*^the number of participants for Insulin, HOMA-IR, and inflammatory markers is as follows: Insulin. CON: >2% gain: 18, ±2% maintain: 44, >2–<5% loss: 27 and 5–20% loss: 17. INT: >2% gain: 14, ±2% maintain: 54, >2–< 5% loss: 43 and 5–20% loss:12. HOMA-IR. CON: >2% gain: 18, ±2% maintain: 42, >2–<5% loss: 26 and 5–20% loss: 17. INT: >2% gain: 14, ±2% maintain: 54, >2–<5% loss: 43 and 5–20% loss: 12. Inflammatory markers. CON: >2% gain: 11, ±2% maintain: 27, >2–<5% loss: 18 and 5–20% loss: 13. INT: >2% gain: 10, ±2% maintain: 34, >2–<5% loss: 29 and 5–20% loss: 9.

**Table 4:** Mean changes in cardiometabolic risk factors by category of weight loss in the maintenance phase.

Cardiometabolic risk markers	Maintenance phase (6 to 12 months)									
	Control					Intervention				
	>2% gain	±2% maintain	>2–<5% loss	5–20% loss	*p* value	>2% gain	±2% maintain	>2–<5% loss	5–20% loss	*p* value
	*n* = 44	*n* = 45	*n* = 16	*n* = 9		*n* = 49	*n* = 57	*n* = 13	*n* = 10	
WC, cm	1.05	−1.09	−4.67^a^	−1.37	**0.001**	0.26	−0.69	−4.59^a^	−3.26	**0.008**
	(−0.32, 2.42)	(−2.44, 0.26)	(−7.00, −2.35)	(−4.59, 1.85)		(−1.13, 1.66)	(−1.98, 0.60)	(−7.31, −1.88)	(−6.41, −0.11)	
FPG^,^ mmol/L	0.17	−0.21	−0.57	0.40	0.003	−0.07	−0.24	−0.31	−0.30	0.584
	(−0.11, 0.46)	(−0.48, 0.07)	(−1.05, −0.09)	(−0.26, 1.05)		(−0.29, 0.14)	(−0.44, −0.04)	(−0.74, 0.11)	(−0.78, 0.19)	
Fasting insulin, *μ*U/mL^*∗*^	1.20	2.10	−1.33	1.26	0.619	1.30	−0.60	0.22	3.74	0.375
	(−1.15, 3.56)	(−0.18, 4.38)	(−6.01, 3.34)	(−4.31, 6.83)		(−0.85, 3.44)	−2.57, 1.38)	(−3.83, 4.26)	(−1.92, 9.40)	
HOMA-IR^*∗*^	0.23	0.47	0.04	−0.24	0.813	0.36	0.31	0.60	−0.61	0.707
	(−0.51, 0.97)	(−0.17, 1.11)	(−1.35, 1.43)	(−1.81, 1.33)		(−0.31, 1.04)	(−0.32, 0.95)	(−0.67, 1.87)	(−2.25, 1.04)	
Triglycerides, mmol/L	0.10	−0.16	0.04	−0.35	0.207	−0.04	−0.18	−0.09	0.13	0.527
	(−0.11, 0.31)	(−0.37, 0.05)	(−0.32, 0.40)	(−0.85, 0.15)		(−0.24, 0.15)	(−0.36, 0.00)	(−0.47, 0.29)	(−0.31, 0.57)	
TC, mmol/L	0.30	0.22	0.26	−0.26	0.414	−0.07	−0.01	−0.33	0.19	0.493
	(0.04, 0.55)	(−0.03, 0.47)	(−0.18, 0.70)	(−0.86, 0.34)		(−0.30, 0.16)	(−0.22, 0.20)	(−0.77, 0.12)	(−0.32, 0.70)	
HDL-C, mmol/L	0.06	−0.07	−0.09	−0.14	**0.018**	−0.03	−0.04	−0.12	−0.07	0.521
	(−0.01, 0.12)	(−0.13, −0.00)	(−0.21, 0.03)	(−0.30, 0.02)		(−0.08, 0.03)	(−0.09, 0.01)	(−0.22, −0.01)	(−0.20, 0.05)	
LDL-C, mmol/L	−0.16	−0.46	−0.45	−0.99	0.051	−0.34	−0.34	−0.89	0.35	0.251
	(−0.41, 0.09)	(−0.71, −0.22)	(−0.88, 0.02)	(−1.58, 0.40)		(−0.56, −0.12)	(−0.55, −0.14)	(−1.32, −0.47)	(−0.14, 0.83)	
TNF-*α*, pg/mL^*∗*^	−5.93	−6.57	−11.60	−7.11	0.050	−6.93	−6.72	−5.50	−6.05	0.939
	(−7.75, −4.11)	(−8.13, −5.00)	(−15.5, −8.05)	(−12.45, −1.65)		(−8.98, −4.87)	(−8.68, −4.76)	(−9.73, −1.25)	(−15.00, −2.91)	
Adiponectin, *μ*g/mL^*∗*^	−0.41	0.89	0.26	0.75	0.886	0.47	−0.91	0.67	1.10	0.287
	(−1.45, 0.62)	(0.00, 1.78)	(−1.76, 2.27)	(−2.33, 3.82)		(−0.68, .61)	(−1.99, 0.17)	(−1.68, 3.03)	(−3.74, 5.94)	
Hs-CRP, *μ*g/mL^*∗*^	0.90	−0.30	−1.99	7.27	0.071	−0.08	2.54	2.89	2.59	0.166
	(−1.11, 12.90)	(−2.03, 1.42)	(−5.91, 1.93)	(1.31, 13.23)		(−1.86, 1.70)	(0.86, 4.22)	(−0.73, 6.51)	(−4.86, 10.03)	

*Note.* Data were presented as estimated marginal mean (95% CI). ^a^Significant difference as relative to >2% weight gain. ^b^Significant difference as relative to ±2% weight maintained. ^*∗*^The number of participants for Insulin, HOMA-IR, and inflammatory markers is as follows: Insulin. CON: >2% gain: 40, ±2% maintain: 42, >2–<5% loss: 11 and 5–20% loss: 8. INT: >2% gain: 46, ±2% maintain: 52, >2–<5% loss: 13 and 5–20% loss: 8. HOMA-IR. CON: >2% gain: 31, ±2% maintain: 41, >2–<5% loss: 10 and 5–20% loss: 8. INT: >2% gain: 46, ±2% maintain: 52, >2–<5% loss: 13 and 5–20% loss: 8. Inflammatory markers. CON: >2% gain: 25, ±2% maintain: 34, >2–<5% loss: 7 and 5–20% loss: 3. INT: >2% gain: 33, ±2% maintain: 37, >2–<5% loss: 8 and 5–20% loss: 2.

## Data Availability

The dataset that supports the findings of this article belongs to the MyBFF@home study. At present, the data are not publicly available but can be obtained from the authors upon reasonable request and with permission from the Director General of Health, Malaysia.

## References

[1] Wang C., Chan J. S. Y., Ren L., Yan J. H. (2016). Obesity reduces cognitive and motor functions across the lifespan. *Neural Plasticity*.

[2] Bastien M., Poirier P., Lemieux I., Després J.-P. (2014). Overview of epidemiology and contribution of obesity to cardiovascular disease. *Progress in Cardiovascular Diseases*.

[3] Garg S. K., Maurer H., Reed K., Selagamsetty R. (2014). Diabetes and cancer: two diseases with obesity as a common risk factor. *Diabetes, Obesity and Metabolism*.

[4] Kitahara C. M., Flint A. J., Berrington De Gonzalez A. (2014). Association between class III obesity (BMI of 40–59 kg/m2) and mortality: a pooled analysis of 20 prospective studies. *PLoS Medicine*.

[5] WHO (2011). *Noncommunicable Diseases Country Profiles 2011*.

[6] Truesdale K. P., Stevens J., Cai J. (2005). The effect of weight history on glucose and lipids. *American Journal of Epidemiology*.

[7] Jensen M. D., Ryan D. H., Apovian C. M. (2014). 2013 AHA/ACC/TOS guideline for the management of overweight and obesity in adults: a report of the American college of cardiology/American heart association task force on practice guidelines and the obesity society. *Circulation*.

[8] Swift D. L., Houmard J. A., Slentz C. A., Kraus W. E. (2018). Effects of aerobic training with and without weight loss on insulin sensitivity and lipids. *PLoS One*.

[9] Wing R. R., Lang W., Wadden T. A. (2011). Benefits of modest weight loss in improving cardiovascular risk factors in overweight and obese individuals with type 2 diabetes. *Diabetes Care*.

[10] Cui Z., Truesdale K. P., Bradshaw P. T., Cai J., Stevens J. (2015). Three-year weight change and cardiometabolic risk factors in obese and normal weight adults who are metabolically healthy: the atherosclerosis risk in communities study. *International Journal of Obesity*.

[11] Welsh P., Cezard G., Gill J. M. (2016). Associations between weight change and biomarkers of cardiometabolic risk in South Asians: secondary analyses of the PODOSA trial. *International Journal of Obesity*.

[12] Wiig Dammann K., Smith C. (2009). Factors affecting low-income women’s food choices and the perceived impact of dietary intake and socioeconomic status on their health and weight. *Journal of Nutrition Education and Behavior*.

[13] Baruth M., Sharpe P. A., Parra-Medina D., Wilcox S. (2014). Perceived barriers to exercise and healthy eating among women from disadvantaged neighborhoods: results from a focus groups assessment. *Women & Health*.

[14] Torres R., Soltero S., Trak M. A. (2016). Lifestyle modification intervention for overweight and obese Hispanic pregnant women: development, implementation, lessons learned and future applications. *Contemporary Clinical Trials Communications*.

[15] Moredich C. A., Kessler T. A. (2014). Physical activity and nutritional weight loss interventions in obese, low-income women: an integrative review. *Journal of Midwifery & Women’s Health*.

[16] Christian J. G., Tsai A. G., Bessesen D. H. (2010). Interpreting weight losses from lifestyle modification trials: using categorical data. *International Journal of Obesity*.

[17] Chang M. W., Brown R., Nitzke S. (2017). Results and lessons learned from a prevention of weight gain program for low-income overweight and obese young mothers: mothers in Motion. *BMC Public Health*.

[18] Walker L. O., Sterling B. S., Latimer L., Kim S.-H., Garcia A. A., Fowles E. R. (2012). Ethnic-specific weight-loss interventions for low-income postpartum women. *Western Journal of Nursing Research*.

[19] Mohamad Nor N. S., Ambak R., Omar M. A. (2016). Methodology of the my body is fit and fabulous st home (Mybff@Home): an intervention study to combat obesity among housewives in Malaysia. *Journal of Womens Health, Issues and Care*.

[20] Matthews D. R., Hosker J. P., Rudenski A. S., Naylor B. A., Treacher D. F., Turner R. C. (1985). Homeostasis model assessment: insulin resistance and ?-cell function from fasting plasma glucose and insulin concentrations in man. *Diabetologia*.

[21] Stevens J., Truesdale K. P., McClain J. E., Cai J. (2006). The definition of weight maintenance. *International Journal of Obesity*.

[22] Zheng Y., Manson J. E., Yuan C. (2017). Associations of weight gain from early to middle adulthood with major health outcomes later in life. *Journal of the American Medical Association*.

[23] Johns D. J., Hartmann-Boyce J., Jebb S. A., Aveyard P., The Behavioural Weight Management Review Group (2016). Weight change among people randomized to minimal intervention control groups in weight loss trials. *Obesity*.

[24] Samuel-Hodge C. D., Johnston L. F., Gizlice Z. (2009). Randomized trial of a behavioral weight loss intervention for low-income women: the weight wise program. *Obesity*.

[25] Ruggiero L., Oros S., Choi Y. K. (2011). Community-based translation of the diabetes prevention program’s lifestyle intervention in an underserved latino population. *The Diabetes Educator*.

[26] Kandula N. R., Dave S., De Chavez P. J. (2015). Translating a heart disease lifestyle intervention into the community: the South Asian heart Lifestyle Intervention (SAHELI) study; a randomized control trial chronic disease epidemiology. *BMC Public Health*.

[27] Teixeira P. J., Silva M. N., Mata J., Palmeira A. L., Markland D. (2012). Motivation, self-determination, and long-term weight control. *International Journal of Behavioral Nutrition and Physical Activity*.

[28] Mohd Zaki N. A., Appannah G., Mohamad Nor N. S. (2018). Impact of community lifestyle intervention on anthropometric parameters and body composition among overweight and obese women: findings from the MyBFF@home study. *BMC Womens Health*.

[29] Balkau B., Picard P., Vol S., Fezeu L., Eschwege E., The Desir Study Group (2007). Consequences of change in waist circumference on cardiometabolic risk factors over 9 Years: data from an epidemiological study on the insulin resistance syndrome (DESIR). *Diabetes Care*.

[30] Balkau B., Deanfield J. E., Després J. P. (2007). International day for the evaluation of abdominal obesity (IDEA). *Circulation*.

[31] Mason C., Foster-Schubert K. E., Imayama I. (2011). Dietary weight loss and exercise effects on insulin resistance in postmenopausal women. *American Journal of Preventive Medicine*.

[32] Swift D. L., Johannsen N. M., Lavie C. J., Earnest C. P., Blair S. N., Church T. S. (2016). Effects of clinically significant weight loss with exercise training on insulin resistance and cardiometabolic adaptations. *Obesity*.

[33] Dow C. A., Thomson C. A., Flatt S. W., Sherwood N. E., Pakiz B., Rock C. L. (2013). Predictors of improvement in cardiometabolic risk factors with weight loss in women. *Journal of the American Heart Association*.

[34] Aucott L., Gray D., Rothnie H., Thapa M., Waweru C. (2011). Effects of lifestyle interventions and long-term weight loss on lipid outcomes—a systematic review. *Obesity Reviews*.

[35] Liyana A. Z., Appannah G., Yazmin S. (2018). Effectiveness of a community-based intervention for weight loss on cardiometabolic risk factors among overweight and obese women in a low socio-economic urban community : findings of the MyBFF @ home. *BMC Womens Health*.

[36] Mohamad Nor N. S., Ambak R., Mohd Zaki N. (2018). An update on obesity research pattern among adults in Malaysia: a scoping review. *BMC Womens Health*.

